# Supplementary material for: Deep mutational scanning reveals functional constraints and antigenic variability of Lassa virus glycoprotein complex
Source: bioRxiv. 2024 Feb 6:2024.02.05.579020. Preprint. [Version 1] doi: 10.1101/2024.02.05.579020 (PMC10871245; doi:10.1101/2024.02.05.579020)
Supplement: Supplement 1 [file NIHPP2024.02.05.579020v1-supplement-1.pdf]

bioRxiv preprint doi: https://doi.org/10.1101/2024.02.05.579020; this version posted February 6, 2024. The copyright holder for this preprint (which was not certified by peer review) is the author/funder, who has granted bioRxiv a license to display the preprint in perpetuity. It is made available under aCC-BY 4.0 International license.

Supplemental Figures

A

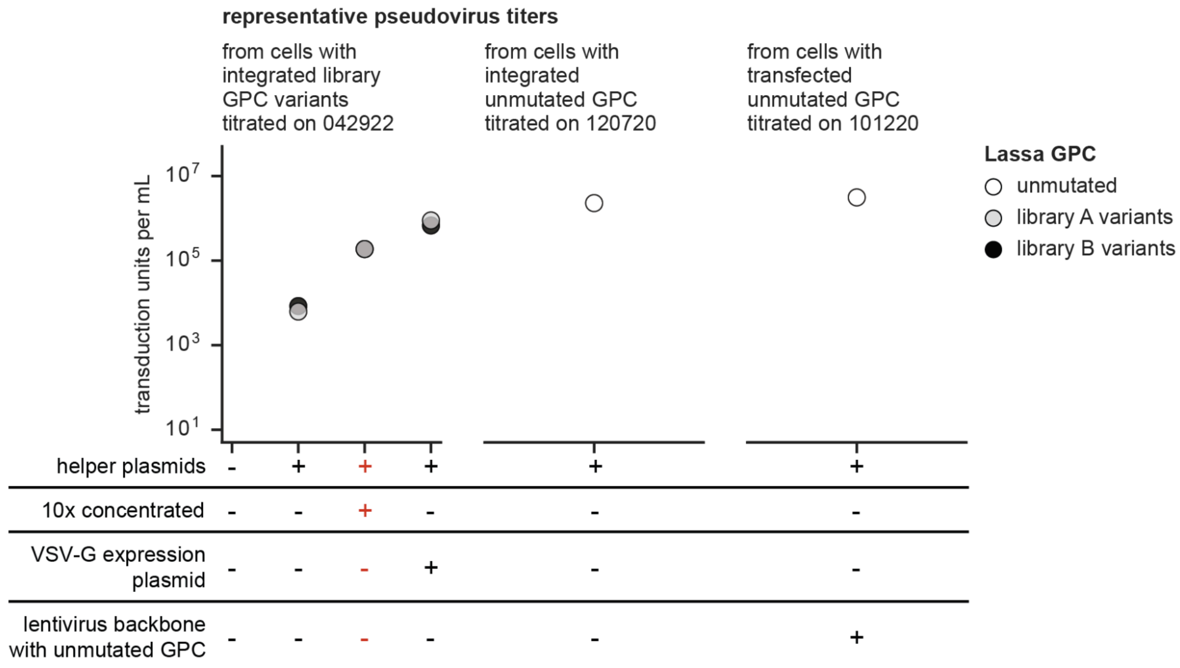

B

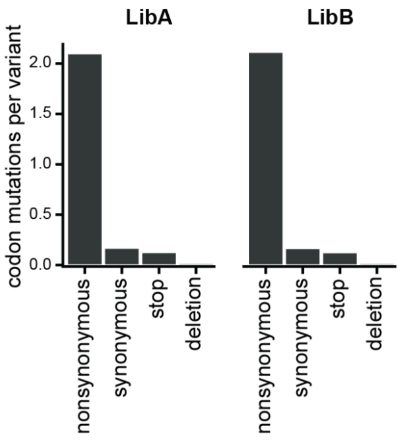

C

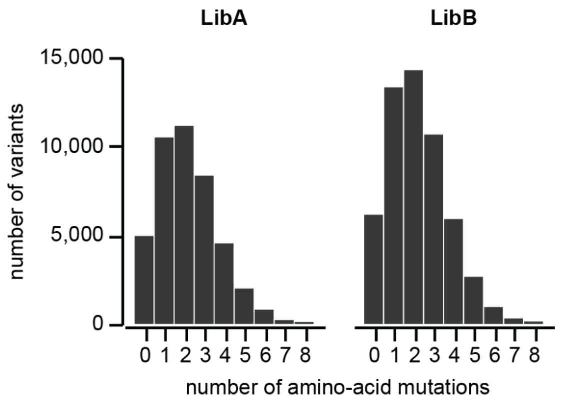

D

Variant library	Number of barcoded variants	Number of unique amino-acid mutations	Amino-acid mutations in the library (% of possible)
Library A	42,460	9,725	99.0
Library B	54,206	9,789	99.7

Figure S1. Characteristics of GPC deep mutational scanning libraries, related to Figure 1

A GPC-pseudotyped lentivirus titers. Pseudotyped viruses were generated from 293T-rtTA cells with a single GPC variant integrated in each cell as depicted in Figure 1B or from transfection of lentivirus backbone containing GPC. Viruses were produced using the indicated conditions and titrated on 293T cells. The GPC-pseudotyped variant viruses used for deep mutational scanning

bioRxiv preprint doi: https://doi.org/10.1101/2024.02.05.579020; this version posted February 6, 2024. The copyright holder for this preprint (which was not certified by peer review) is the author/funder, who has granted bioRxiv a license to display the preprint in perpetuity. It is made available under aCC-BY 4.0 International license.

were generated under the conditions highlighted in red. The VSV-G condition was used to generate VSV-G-pseudotyped variant viruses, which were used to assess the composition of mutations present in the variant libraries. **B** Average number of codon mutations per barcoded variant for each GPC-pseudotyped variant library. **C** Distribution of the number of amino-acid mutations in each GPC variant for each library. **D** The total number of barcoded variants, unique amino-acid mutations, and percentage of possible amino-acid mutations for each GPC-pseudotyped variant library.

bioRxiv preprint doi: https://doi.org/10.1101/2024.02.05.579020; this version posted February 6, 2024. The copyright holder for this preprint (which was not certified by peer review) is the author/funder, who has granted bioRxiv a license to display the preprint in perpetuity. It is made available under aCC-BY 4.0 International license.

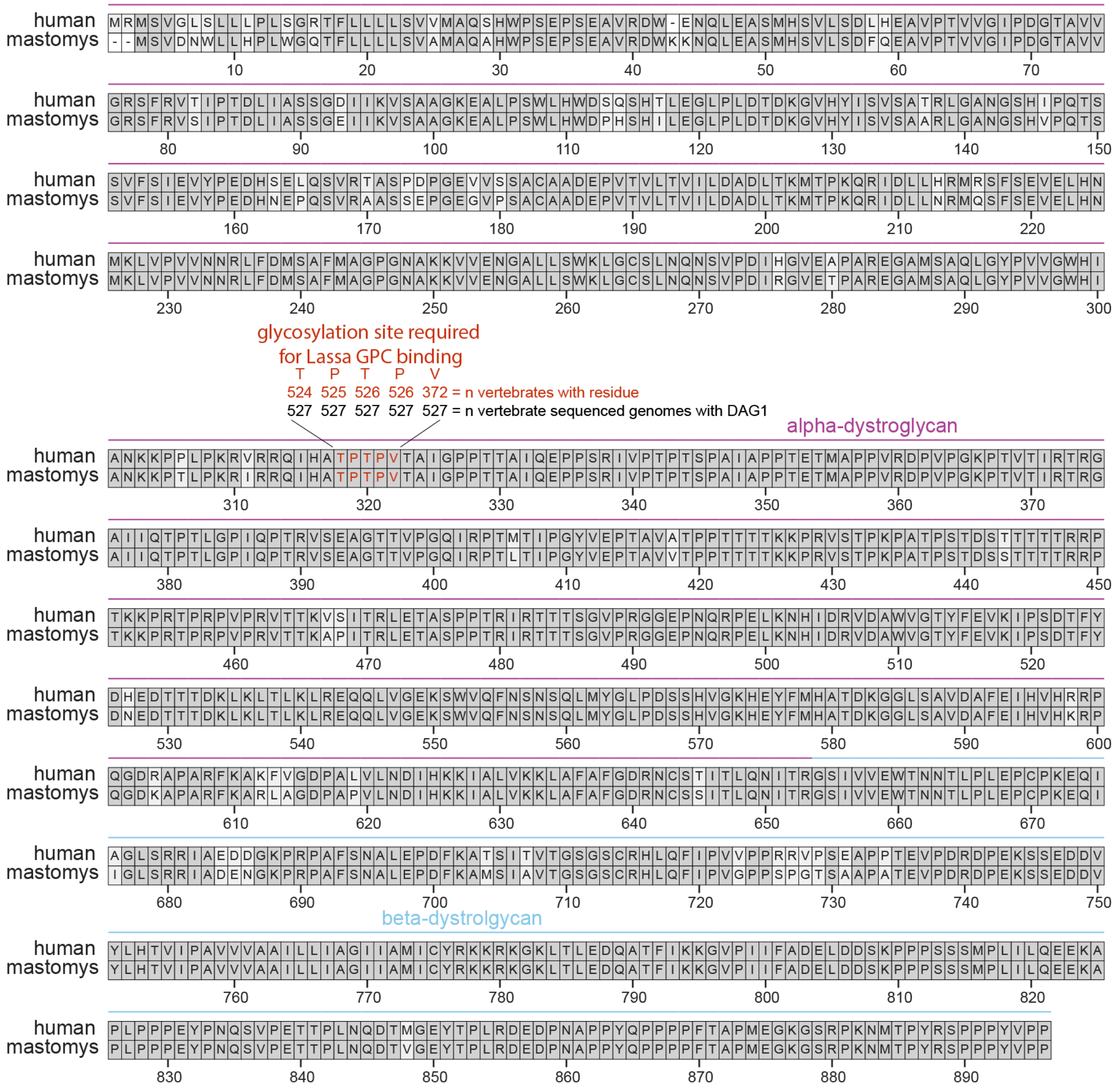

Figure S2. Human and mastomys DAG1 sequence alignment, related to Figure 2

Protein sequence alignment of human and mastomys DAG1. The alpha-dystroglycan (α-DG) and beta-dystroglycan (β-DG) subunits are highlighted in purple and blue, respectively. The TPTPV glycosylation motif required for laminin and Lassa virus GPC binding[61] is highlighted in red with the conservation across all vertebrate sequences shown in the text. The overall conservation between human and mastomys α-DG at the protein level is 93%.

bioRxiv preprint doi: https://doi.org/10.1101/2024.02.05.579020; this version posted February 6, 2024. The copyright holder for this preprint (which was not certified by peer review) is the author/funder, who has granted bioRxiv a license to display the preprint in perpetuity. It is made available under aCC-BY 4.0 International license.

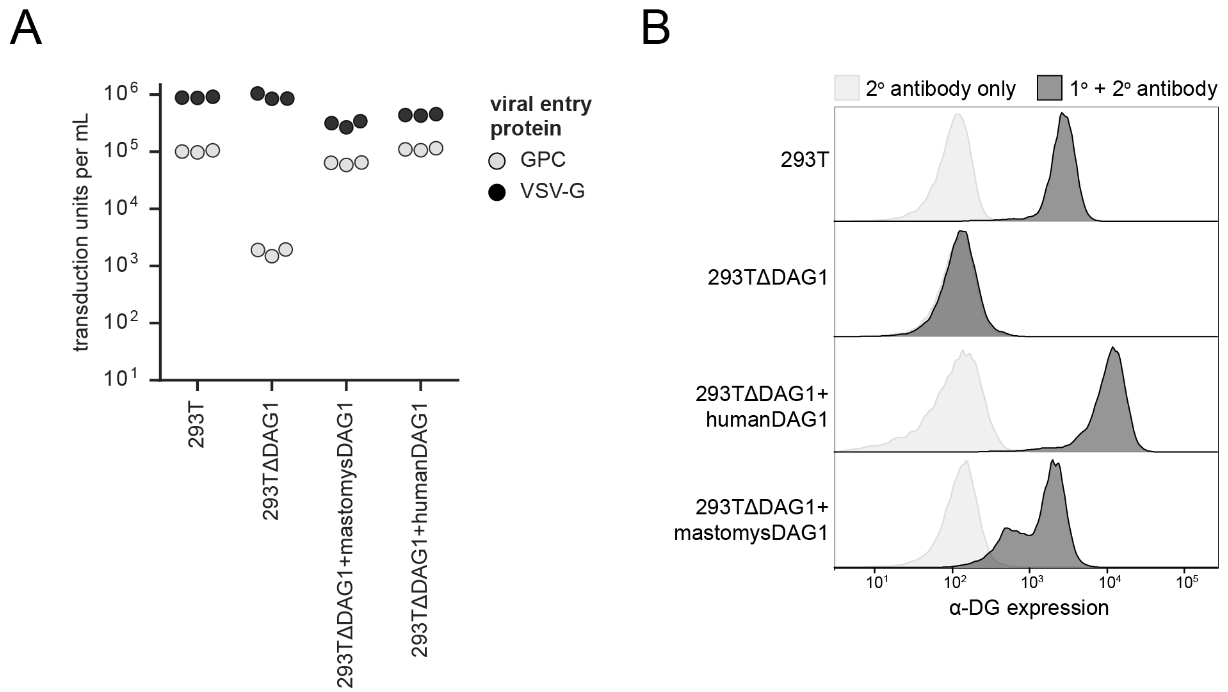

Figure S3. GPC mediates effective entry into cells expressing human or mastomys DAG1 but not cells lacking DAG1, related to Figure 2

A Titers of VSV-G or Lassa GPC pseudotyped lentivirus in different cell lines. 293T cells natively express human α-DG from their *DAG1* gene, 293TΔDAG1 have had this gene disrupted and so do not express α-DG,[27] and 293TΔDAG1+mastomysDAG1 / 293TΔDAG1+humanDAG1 cells are variants of 293TΔDAG1 that we stably transduced with the mastomys or human *DAG1* gene. VSV-G-mediated cell entry is independent of *DAG1* expression since VSV-G uses a different receptor, but GPC-mediated cell entry is only efficient in the presence of α-DG expression from the *DAG1* gene. **B** α-DG expression for different cell lines. Surface α-DG expression was measured using flow cytometry with an antibody against dystroglycan and the histograms show the distribution of expression over a population of cells. See “Methods” for details.

bioRxiv preprint doi: https://doi.org/10.1101/2024.02.05.579020; this version posted February 6, 2024. The copyright holder for this preprint (which was not certified by peer review) is the author/funder, who has granted bioRxiv a license to display the preprint in perpetuity. It is made available under aCC-BY 4.0 International license.

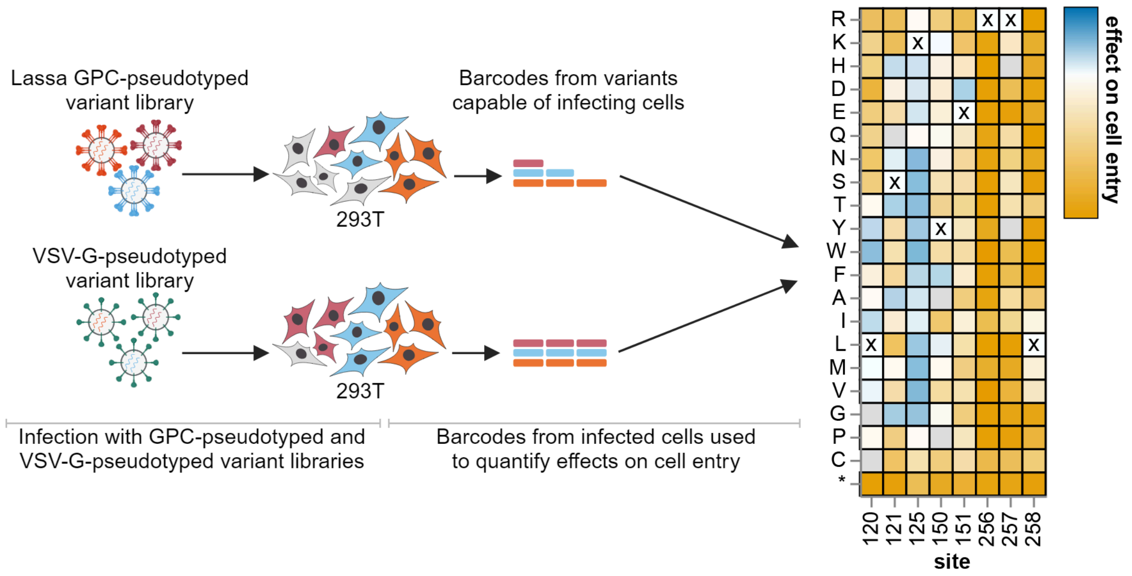

Figure S4. Workflow for measuring how mutations affect cell entry, related to Figure 2

Cells are infected with either the GPC-pseudotyped variant library or the VSV-G-pseudotyped library, and variant barcodes are sequenced from the infected cells. All variants infect cells when VSV-G is present, but only variants with functional GPC infect cells when VSV-G is not present. The log enrichment or depletion of variants in the GPC-pseudotyped condition relative to the VSV-G-pseudotyped condition quantifies the functional capability of variants to enter cells. Individual mutation effects are inferred from the single- and multi-mutant variants using a global epistasis model.[70]

bioRxiv preprint doi: https://doi.org/10.1101/2024.02.05.579020; this version posted February 6, 2024. The copyright holder for this preprint (which was not certified by peer review) is the author/funder, who has granted bioRxiv a license to display the preprint in perpetuity. It is made available under aCC-BY 4.0 International license.

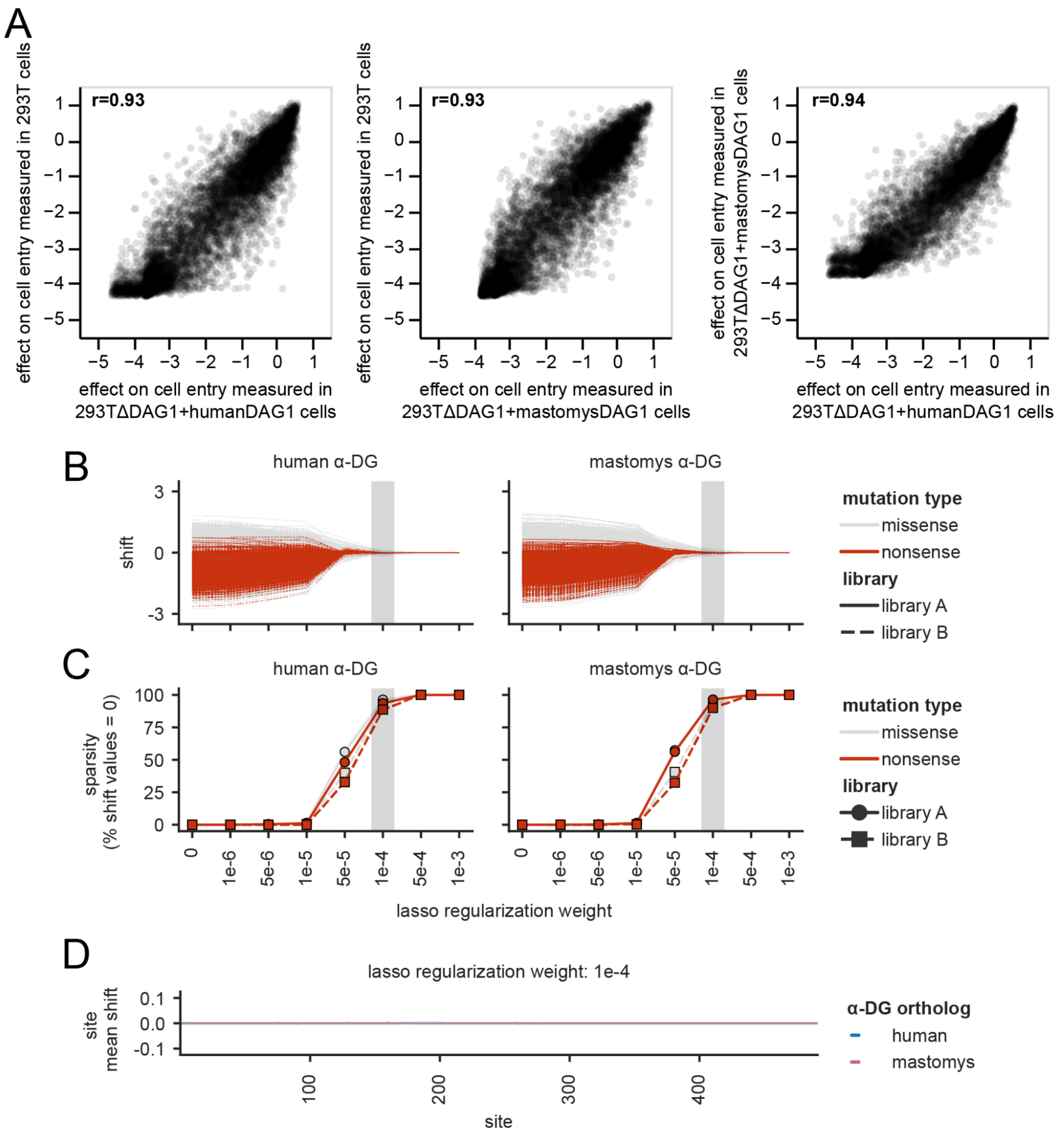

Figure S5. Effects of GPC mutations on entry into 293T cells expressing human or mastomys α-DG, related to Figure 2

A Correlation of mutation effects on cell entry measured in 293T, 293TΔDAG1+humanDAG1, and 293TΔDAG1+mastomysDAG1 cells. See https://dms-vep.org/LASV_Josiah_GP_DMS/htmls/DAG1_ortholog_correlations.html for an interactive version of correlation plots that allow you to mouse over points for amino-acid identities. The Pearson correlation (r) is indicated. For interactive plots of the effects of all mutations on entry, see the following links: for 293T cells

bioRxiv preprint doi: https://doi.org/10.1101/2024.02.05.579020; this version posted February 6, 2024. The copyright holder for this preprint (which was not certified by peer review) is the author/funder, who has granted bioRxiv a license to display the preprint in perpetuity. It is made available under aCC-BY 4.0 International license.

(https://dms-vep.org/LASV_Josiah_GP_DMS/htmls/293T_entry_func_effects.html), for 293TΔDAG1+humanDAG1 (https://dms-vep.org/LASV_Josiah_GP_DMS/htmls/human_293T_entry_func_effects.html), and for 293TΔDAG1+mastomysDAG1 (https://dms-vep.org/LASV_Josiah_GP_DMS/htmls/mastomys_293T_entry_func_effects.html). **B-D** Analysis of possible shifts in mutation effects in cells expressing human versus mastomys α-DG as assessed using the more sophisticated algorithm implemented in the *multidms* software package (https://github.com/matsengrp/multidms).[64] **B** Shifts in mutation effects on cell entry measured on 293TΔDAG1+human and 293TΔDAG1+mastomys cell lines relative to 293T cells. Each line tracks the shift value for a single mutation across different lasso weight regularization values. Red lines are for all nonsense mutations and gray lines are for all missense mutations. Individual library measurements are shown in solid and dashed lines. **C** The sparsity (i.e., percent shift values equal to zero) across different lasso weight regularization values. Lines are colored as indicated in **B**. **D** Per site average shift value for mutations measured on 293TΔDAG1+human and 293TΔDAG1+mastomys cell lines for a lasso weight of 1e-4. This is a reasonable lasso weight because it regularizes apparent shifts for stop codons (which should not have human versus mastomys specific effects) to zero. At this lasso weight, the flat line in this plot shows that there are no sites with an appreciable shift in mutation effects.

bioRxiv preprint doi: https://doi.org/10.1101/2024.02.05.579020; this version posted February 6, 2024. The copyright holder for this preprint (which was not certified by peer review) is the author/funder, who has granted bioRxiv a license to display the preprint in perpetuity. It is made available under aCC-BY 4.0 International license.

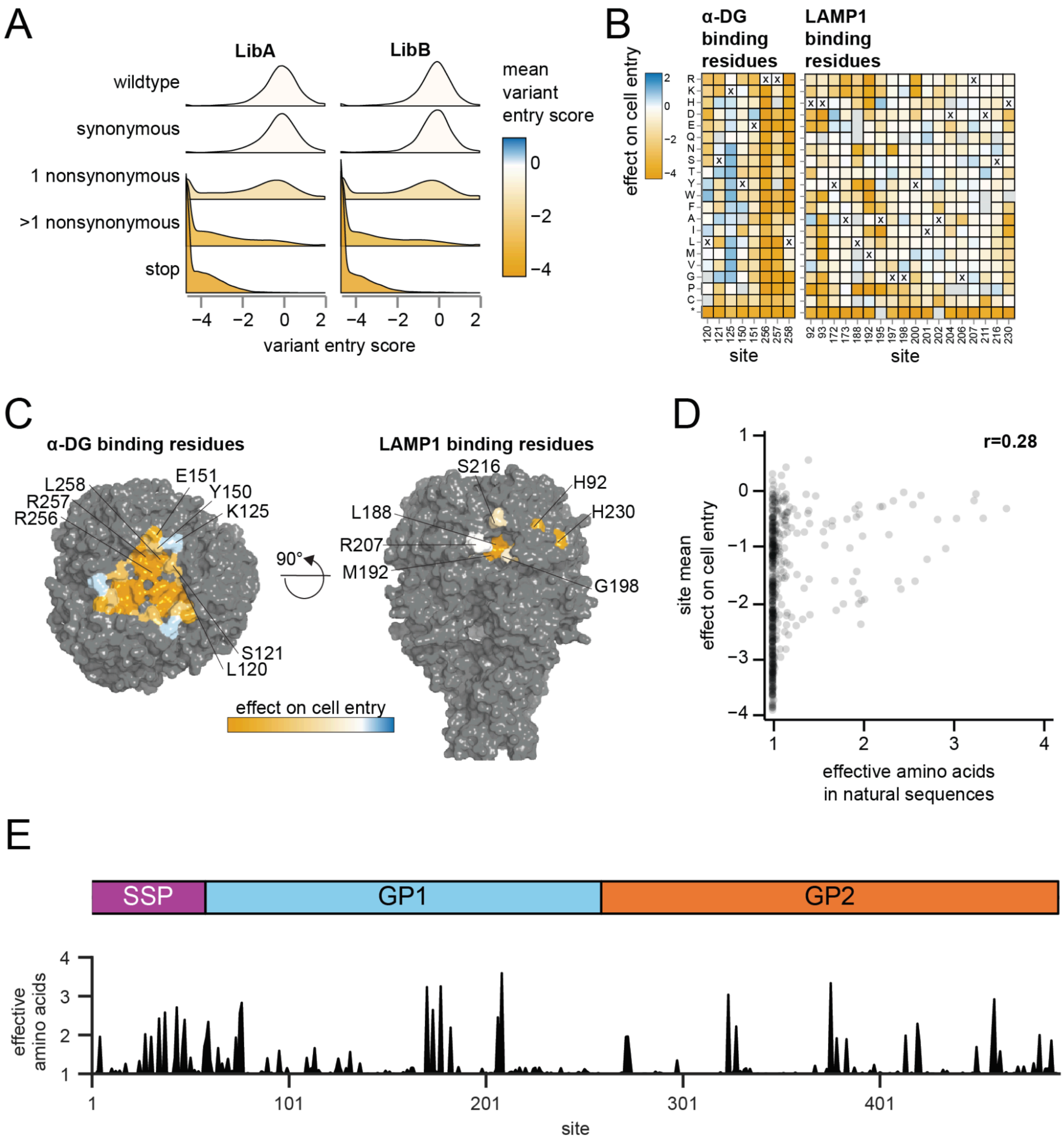

Figure S6. Effects of mutations on cell entry for specific GPC regions and in comparison to natural sequence diversity, related to Figure 2

A Distributions of deep mutational scanning cell-entry scores for variants with different mutant types. The cell-entry score is the log ratio of the variant frequency relative to the unmutated parental strain in the GPC-pseudotyped library versus the VSV-G-pseudotyped library. Negative and positive scores indicate decreased and increased GPC-mediated cell entry relative to unmutated GPC, respectively. Distributions are colored by mean cell-entry score. **B** Heatmaps of effects on cell entry of individual mutations at key sites for host receptor binding.

bioRxiv preprint doi: https://doi.org/10.1101/2024.02.05.579020; this version posted February 6, 2024. The copyright holder for this preprint (which was not certified by peer review) is the author/funder, who has granted bioRxiv a license to display the preprint in perpetuity. It is made available under aCC-BY 4.0 International license.

Sites involved in α-DG[52] binding and sites suggested to be involved in LAMP1[71,72] binding are shown. **C** Surface representation of Lassa GPC colored by per site average amino-acid effect on cell entry for key sites for α-DG and LAMP1 binding (PDB: 7PUY). **D** Correlation of per site average effects of amino-acid mutations on cell entry measured by deep mutational scanning and natural sequence diversity as described in **E**. **E** Top shows a schematic of Lassa GPC with the stable signal peptide (SSP), glycoprotein 1 (GP1), and glycoprotein 2 (GP2) highlighted. Below is a plot of natural sequence diversity across an alignment of all high quality Lassa GPC sequences. Diversity is quantified as the effective number of amino acids at a site.[94]

bioRxiv preprint doi: https://doi.org/10.1101/2024.02.05.579020; this version posted February 6, 2024. The copyright holder for this preprint (which was not certified by peer review) is the author/funder, who has granted bioRxiv a license to display the preprint in perpetuity. It is made available under aCC-BY 4.0 International license.

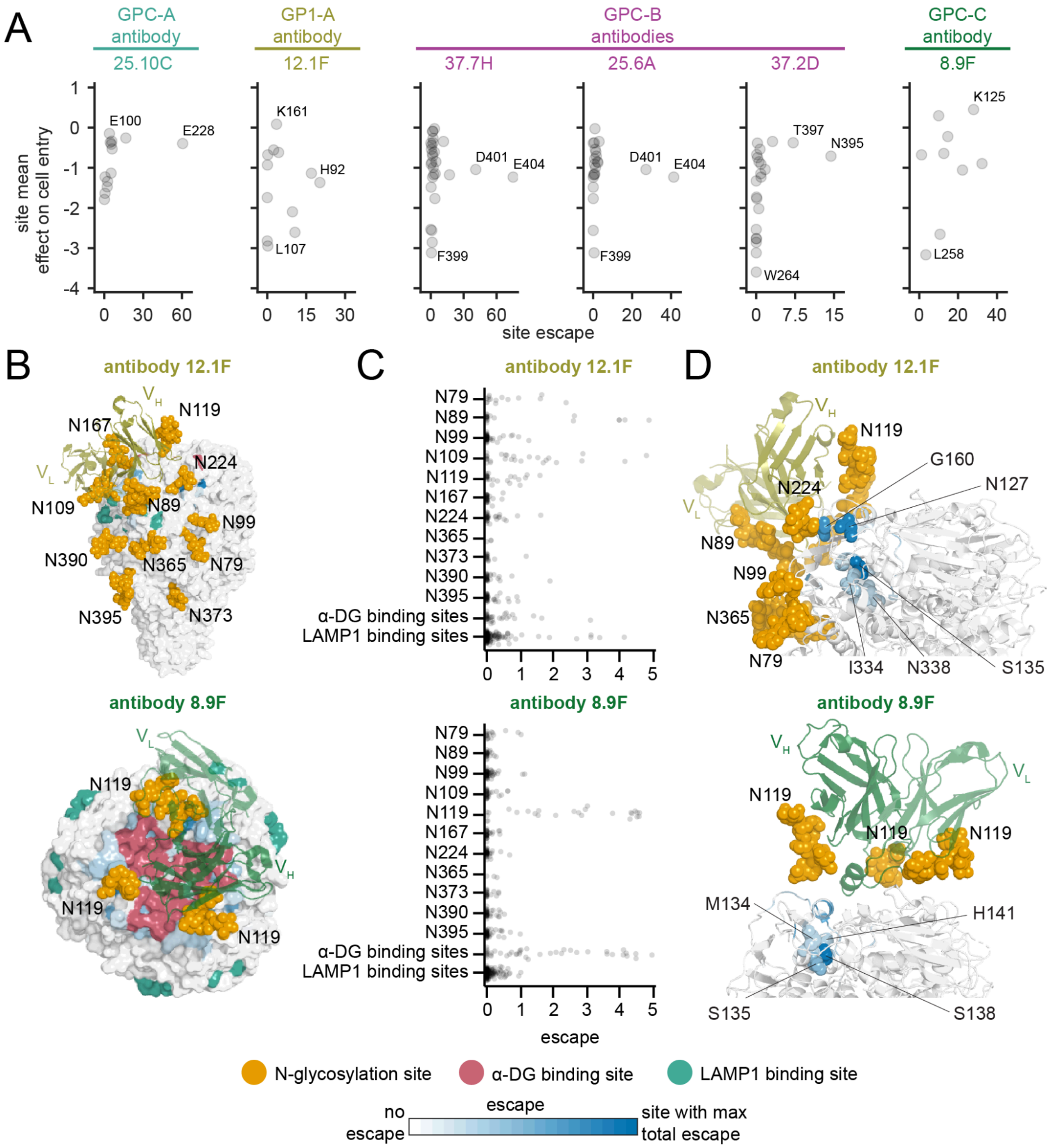

Figure S7. Effects of mutations on antibody escape in comparison to effects on cell entry and for specific GPC sites, related to Figures 4 and 5

A Comparison of per site average effects of amino-acid mutations on cell entry and per site summed effects of mutations on antibody escape for all sites that contact the antibody (within 4 Å). For instance, site L258 (which contacts antibody 8.9F) has low mutational tolerance, which may explain why it is not measured to cause escape as our assay can only quantify escape for mutations tolerated for GPC-mediated cell entry. On the other hand, site E100 (which contacts antibody 25.10C) has low escape even though mutations there are well tolerated, suggesting it is

bioRxiv preprint doi: https://doi.org/10.1101/2024.02.05.579020; this version posted February 6, 2024. The copyright holder for this preprint (which was not certified by peer review) is the author/funder, who has granted bioRxiv a license to display the preprint in perpetuity. It is made available under aCC-BY 4.0 International license.

simply not important for antibody binding. The antibody escape maps are grouped by antibody epitope classification of Robinson et al.[28] See https://dms-vep.org/LASV_Josiah_GP_DMS/htmls/antibody_escape_vs_func_effect.html for an interactive version of this plot. **B** Surface representation of Fab-bound GPC colored by site escape as measured in deep mutational scanning, with the Fab shown in a colored cartoon representation. Antibody 12.1F inhibits receptor binding and relies on five N-linked glycans (N89, N109, N119, N167, and N224) to bind to a single monomer of GPC[31,78]; therefore, mutations to glycosylation motifs (N-X-S/T, X≠P) and to receptor binding sites escape antibody 12.1F neutralization as shown in **C**. Similarly, antibody 8.9F inhibits α-DG binding and relies on the N-linked glycan N119 to bind across the three monomers of GPC[31]; therefore, mutations to α-DG binding sites and to the N119 glycosylation motif escape 8.9F neutralization as shown in **C**. Blue indicates the GPC site with the most escape from that antibody, and white indicates sites with no escape. N-linked glycans (orange spheres), α-DG binding sites (pink), and LAMP1 binding sites (turquoise) are highlighted. The Fab bound antibody structures shown here come from prior cryo-EM structures.[31] **C** Effects of mutations on antibody escape for N-linked glycosylation sites (N-X-S/T, X≠P) and receptor binding sites. Mutations affecting glycans that are important for antibody binding (e.g., antibody 8.9F relies on N-linked glycan N119) tend to lead to antibody escape. Each point represents a different amino-acid mutation. **D** Zoomed in view of cartoon representation of Fab-bound GPC colored by site escape as measured in deep mutational scanning, with the Fab shown in a colored cartoon. Antibody escape sites that are more distally located from the antibody are highlighted by spheres. For instance, site S135 is neither an antibody 12.1F contact nor part of a glycosylation motif that is important for 12.1F binding, but mutations to this site lead to antibody escape.

bioRxiv preprint doi: https://doi.org/10.1101/2024.02.05.579020; this version posted February 6, 2024. The copyright holder for this preprint (which was not certified by peer review) is the author/funder, who has granted bioRxiv a license to display the preprint in perpetuity. It is made available under aCC-BY 4.0 International license.

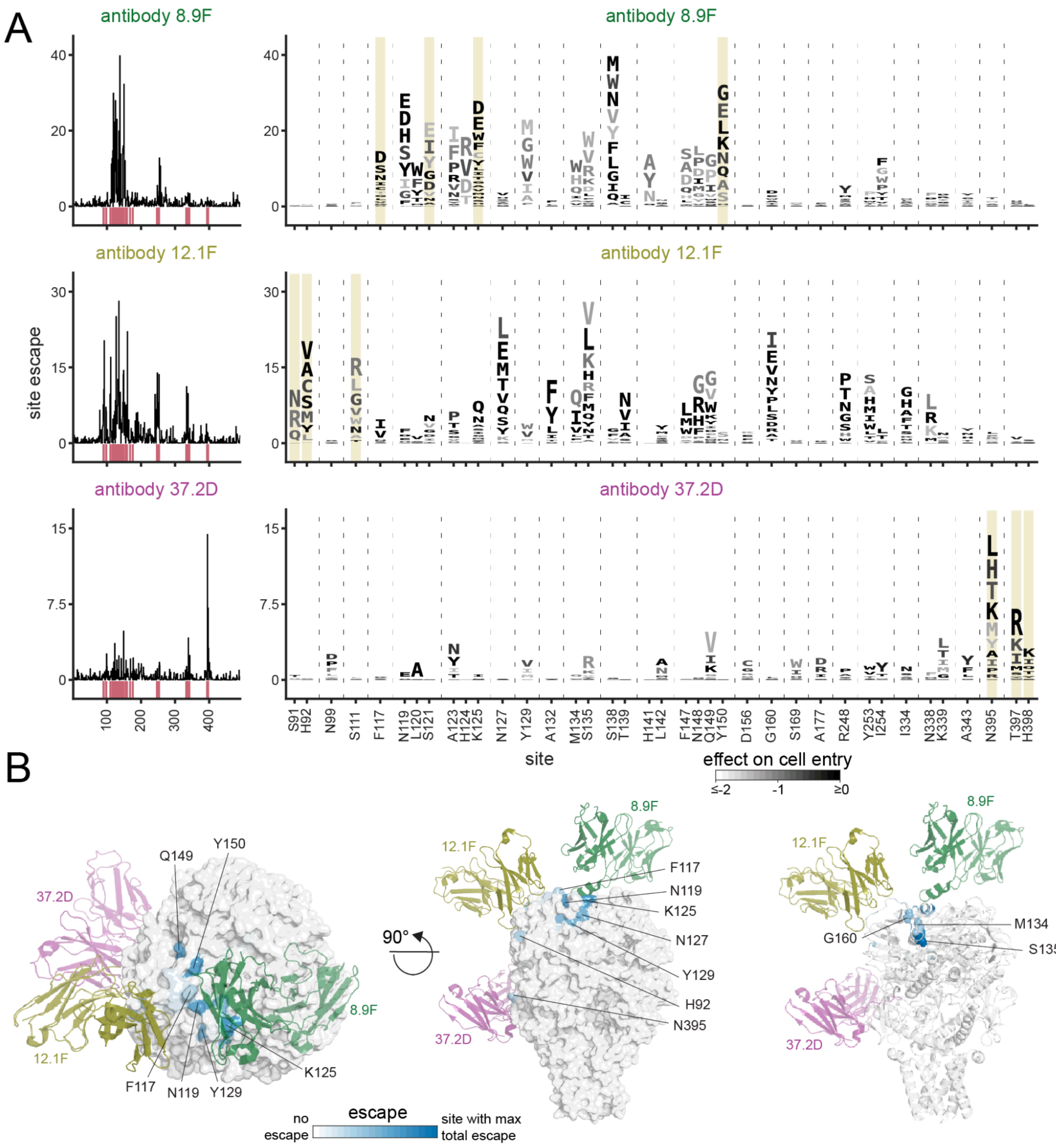

Figure S8. Effects of mutations on antibody escape for the antibodies that comprise the antibody cocktail Arevirumab-3, related to Figures 4 and 5

A Escape maps for the antibodies (8.9F, 12.1F, and 37.2D) that comprise the antibody cocktail Arevirumab-3. Line plots show site summed effects of all escape mutations at a site as measured in the deep mutational scanning. The top 15 escape sites across the three antibodies are highlighted pink below the line plot and shown in logo plots where the height of each letter indicates escape caused by that mutation. Letters are colored by mutational effects on cell entry in the absence of antibody, with mutations that impair entry shown in lighter gray. Sites that

bioRxiv preprint doi: https://doi.org/10.1101/2024.02.05.579020; this version posted February 6, 2024. The copyright holder for this preprint (which was not certified by peer review) is the author/funder, who has granted bioRxiv a license to display the preprint in perpetuity. It is made available under aCC-BY 4.0 International license.

contact the antibody (within 4 Å) are highlighted in yellow. **B** Surface representation of Fab-bound GPC colored by site escape averaged across the three Arevirumab-3 antibodies (8.9F, 12.1F, and 37.2D) as measured in deep mutational scanning, with the Fab of the antibodies shown in colored cartoon representations. Because GPC is a homo-trimer, escape is colored only on sites in the monomer that is closest to the antibodies shown. Blue indicates the GPC site with the most escape from that antibody, and white indicates sites with no escape. The Fab bound antibody structures shown here come from prior cryo-EM structures.[31] See beginning of "Methods" for links to more detailed interactive versions of each escape map.

bioRxiv preprint doi: https://doi.org/10.1101/2024.02.05.579020; this version posted February 6, 2024. The copyright holder for this preprint (which was not certified by peer review) is the author/funder, who has granted bioRxiv a license to display the preprint in perpetuity. It is made available under aCC-BY 4.0 International license.

A

Natural strain	S segment accession	Country from which sequence was isolated	Host from which sequence was isolated	Date of collection
LM395	KM822115	Sierra Leone	Mastomys natalensis	2009-XX-XX
ISTH1024	MH157037	Nigeria	Homo sapiens	2018-02-14
GA391	OL774861	Nigeria	Homo sapiens	1977-XX-XX
IRR007	MK107922	Nigeria	Homo sapiens	2016-01-18

B

Codon position	Forward read bases	Reverse read bases
1	15 A, 7 G	3 A, 9 G
2	23 A	13 A
3	24 C	13 C

Figure S9. Natural strain GPCs used for neutralization assays, related to Figures 6 and 7

A Additional metadata for natural strain GPCs used for experiments. **B** Short-read sequencing data for LM395 strain at codon position 89. The columns indicate read counts in the forward and reverse strands. The AAC codon encodes N (the identity in Josiah) and the GAC codon encodes D (the polymorphic mutant identity in LM395 strain at position 89). See "Methods" for more details on analysis.

bioRxiv preprint doi: https://doi.org/10.1101/2024.02.05.579020; this version posted February 6, 2024. The copyright holder for this preprint (which was not certified by peer review) is the author/funder, who has granted bioRxiv a license to display the preprint in perpetuity. It is made available under aCC-BY 4.0 International license.

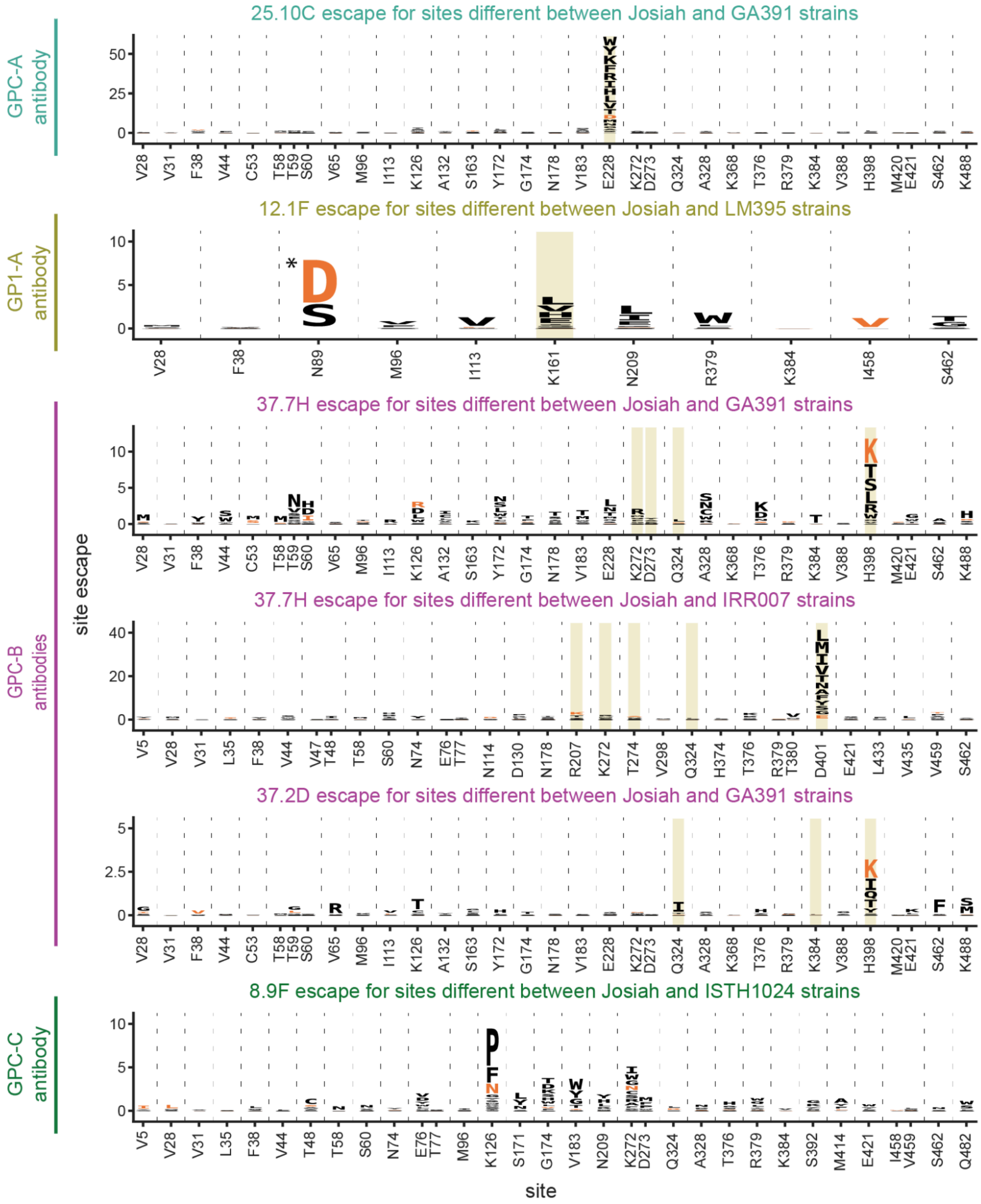

**LM395 strain is polymorphic at site 89 (D/N) and the only strain with a mutation at site 89*

Figure S10. Escape maps for all sites that differ between Josiah and natural strain GPCs, related to Figure 6 and 7

Antibody escape maps for all amino-acid sites that differ between the Josiah GPC strain and the natural strain GPC chosen for validation. This plot differs from Figure 7A as it shows all sites that differ between the GPCs, rather than just the top 10 escape sites. As in Figure 7A, the height of the letter corresponds to the strength of escape, and the amino acid present in the natural isolate is colored orange. Sites that contact the antibody (within 4 Å of antibody) are highlighted yellow. The antibody escape maps are grouped by antibody epitope classification of Robinson et

bioRxiv preprint doi: https://doi.org/10.1101/2024.02.05.579020; this version posted February 6, 2024. The copyright holder for this preprint (which was not certified by peer review) is the author/funder, who has granted bioRxiv a license to display the preprint in perpetuity. It is made available under aCC-BY 4.0 International license.

al.[28] *N89D is marked because the LM395 strain with the N89D mutation is polymorphic at site 89 (Figure S9B)[17] and is the only strain with a mutation at site 89.
